# Hepatoprotective Effect of the Ethanol Extract of *Illicium henryi* against Acute Liver Injury in Mice Induced by Lipopolysaccharide

**DOI:** 10.3390/antiox8100446

**Published:** 2019-10-01

**Authors:** Md Sodrul Islam, Hui Yu, Lingyan Miao, Zhaoying Liu, Yanfei He, Hongxiang Sun

**Affiliations:** 1 College of Animal Sciences, Zhejiang University, Hangzhou 310058, China; msislam@bsmrau.edu.bd (M.S.I.); 645355598@zju.edu.cn (L.M.); 11717039@zju.edu.cn (Y.H.); 2 Departments of Physiology and Pharmacology, Bangabandhu Sheikh Mujibur Rahman Agricultural University, Gazipur 1706, Bangladesh; 3 College of Veterinary Medicine, Hunan Agricultural University, Changsha 410128, China; 17605032171@sina.cn (H.Y.); liu_zhaoying@hunau.edu.cn (Z.L.)

**Keywords:** *Illicium henryi*, lipopolysaccharide, acute liver injury, TLR4, NF-κB, Nrf2

## Abstract

The root bark of *Illicium henryi* has been used in traditional Chinese medicine to treat lumbar muscle strain and rheumatic pain. Its ethanol extract (EEIH) has been previously reported to attenuate lipopolysaccharide (LPS)-induced acute kidney injury in mice. The present study aimed to evaluate the in vitro antioxidant activities and in vivo protective effects of EEIH against LPS-induced acute liver injury (ALI) in mice as well as explore its molecular mechanisms. The mice were injected intraperitoneally (*i.p.*) with EEIH at the doses of 1.25, 2.5, and 5.0 mg/kg every day for 5 days. One hour after the last administration, the mice were administered *i.p.* with LPS (8 mg/kg). After fasting for 12 h, blood and liver tissues were collected to histopathological observation, biochemical assay, enzyme-linked immunosorbent assay (ELISA), quantitative real-time polymerase chain reaction (qRT-PCR), and Western blot analyses. EEIH possessed 2,2-diphenyl-1-picrylhydrazil (DPPH) and 2,2′-azino-bis-(3-ethylbenzothiozoline-6-sulfonic acid) disodium salt (ABTS) radical scavenging activities and ferric-reducing antioxidant capacity in vitro. The histopathological examination, serum biochemical analysis, and liver myeloperoxidase (MPO) activity showed that EEIH pretreatment alleviated LPS-induced liver injury in mice. EEIH significantly dose-dependently decreased the mRNA and protein expression levels of inflammatory factors TNF-α, IL-1β, IL-6, and COX-2 in liver tissue of LPS-induced ALI mice via downregulating the mRNA and protein expressions of toll-like receptor 4 (TLR4) and inhibiting the phosphorylation of nuclear factor-κB (NF-κB) p65. Furthermore, EEIH markedly ameliorated liver oxidative and nitrosative stress burden in LPS-treated mice through reducing the content of thiobarbituric acid reactive substances (TBARS), inducible nitric oxide synthase (iNOS), and nitric oxide (NO) levels, restoring the decreased superoxide dismutase (SOD) and reduced glutathione (GSH) levels, and up-regulating nuclear factor erythroid 2 related factor 2 (Nrf2). These results demonstrate that EEIH has protective effects against ALI in mice via alleviating inflammatory response, oxidative and nitrosative stress burden through activating the Nrf2 and suppressing the TLR4/NF-κB signaling pathways. The hepatoprotective activity of EEIH might be attributed to the flavonoid compounds such as catechin (**1**), 3′,4′,7-trihydroxyflavone (**2**), and taxifolin (**7**) that most possibly act synergistically.

## 1. Introduction

The liver plays a crucial role in clearing pathogens and immunological reaction [1] and is vulnerable to chemical toxic compounds that cause acute liver injury (ALI) [2]. Sustained liver injury can result in liver fibrosis and liver dysfunction [3]. Lipopolysaccharide (LPS) was proved to induce liver injury [4] and ultimately result in sepsis [5]. Sepsis is a severe organ dysfunction due to an unregulated host reaction to infection and is a global public health concern with restricted therapeutic choices [6]. The most common complication of sepsis is liver dysfunction with an incidence rate up to 40% [7]. In the septic patients associated with liver dysfunction, the mortality rate reaches up to 54–68% [5]. Sepsis is one of the main symptoms of ALI [8] and the patient ultimately needs liver transplantation in clinic [9]. Therefore, the continued search for novel, safe, and effective drugs is of utmost importance.

Single dose of intraperitoneal administration of LPS has usually been used as extensive approval in the clinically applicable model of serious liver injury [10]. LPS could activate toll-like receptor 4 (TLR4) in hepatocytes and kupffer cells [11], and then induce the phosphorylation of nuclear factor-κB (NF-κB) and the secretions of the pro-inflammatory cytokines such as IL-6, TNF-α, and IL-1β, thereby exacerbate liver inflammation injury [12]. Moreover, LPS-induced hepatocellular injury also elicits oxidative and nitrosative stress to result in an increase of oxidants like reactive oxygen (ROS) and nitrogen species and reduction of endogenous antioxidants such as superoxide dismutase (SOD) and glutathione (GSH) and elevation of malondialdehyde (MDA), which is implied in the pathophysiology of ALI [13,14]. Nuclear factor E2 related factor 2 (Nrf2), a main controller of the cellular redox status, prevents as the major defense mechanism from LPS-induced hepatocellular oxidative stress [15]. Thus, suppressing inflammation and oxidative stress might be an approach to prevent and control ALI.

Medicinal plants have been a dynamic area of interest to remedy liver disorders due to safety and potency [16]. *Illicium henryi* Diels (Illiciaceae), commonly known as Chinese Anise Tree or hardwood crab, is an indigenous evergreen shrub or tree, and mostly distributed at 300–2200 m altitude in the hills or mountain regions of Fujian, Anhui, Guangdong, Gansu, Guangxi, Henan, Guizhou, Hubei, Hunan, Jiangxi, Yunnan, Shanxi, and Sichuan provinces in China [17]. *I. henryi* has been officially recorded in the “Compendium of Materia Medica” of the Ming Dynasty and used in traditional Chinese medicine for a long time. Its root bark was used for alleviating pain and swelling, removing phlegm, promoting blood circulation, and dispelling pathogenic wind, cold and dampness in traditional Chinese medicine [17]. *I. henryi* was reported to possess anti-inflammatory, antioxidant [18], anti-hepatitis B virus (HBV) and anti-HIV activities [19]. Phytochemical studies showed that the root bark of *I. henryi* contained flavonoids, neolignans [20], sesquiterpene [21], lignans [22], and prenylated C6–C3 compounds [23]. In our previous study, the ethanol extract of *I. henryi* root bark (EEIH) was found to attenuate LPS-induced acute kidney injury in mice [18]. In this study, EEIH was evaluated for in vitro antioxidant activities and in vivo protective effects against LPS-induced ALI in mice as well as explored its mechanisms associated with the Nrf2 activation and TLR4/NF-κB signaling inhibition in oxidative stress and inflammation.

## 2. Materials and Methods

### 2.1. Chemical and Reagents

LPS (from *Escherichia coli* 055:B5), vitamin C, 2,2-diphenyl-1-picrylhydrazyl (DPPH), and 2,2′-azino-bis-(3-ethylbenzothiazoline-6-sulphonic acid) diammonium salt (ABTS) were purchased from Sigma-Aldrich Chemical Co., St. Louis, MO, USA. TNF-α, IL-1β, and IL-6 ELISA kits were from Boster Biological Technology Co. Ltd., Wuhan, Hubei, China. Glutathione (GSH), malondialdehyde (MDA), myeloperoxidase (MPO), nitric oxide (NO), and superoxide dismutase (SOD) assay kits were from Jiancheng Bioengineering Institute, Nanjing, Jiangsu, China. TRIzol reagent was from Invitrogen, Carlsbad, CA, USA. Revert Aid™ M-MLV reverse transcriptase was from Fermentas, Amherst, NY, USA. Ribonuclease inhibitor and oligo(dT)18 were from Sangon Biotech (Shanghai) Co. Ltd., Shanghai, China. FastStart Universal SYBR Green Master (ROX) was from Roche Diagnostics, Indianapolis, IN, USA. BCA protein assay kit, RIPA lysis buffer, 10% sodium dodecyl sulfate-polyacrylamide gel electrophoresis (SDS-PAGE), horseradish-peroxidase (HRP)-conjugated goat anti-rabbit and anti-mouse IgG (H+L), and BeyoECL Star kit were from Beyotime Biotechnology, Shanghai, China. Phosphatase inhibitor cocktail and protease inhibitor cocktail were from Bimake, Houston, TX, USA. Anti-rabbit NF-κB p65 (C-20) and TLR4 polyclonal antibodies were from Santa Cruz Biotechnology, Dallas, TX, USA. Anti-mouse actin monoclonal antibody and anti-rabbit phospho-NF-κB p65 (Ser536) polyclonal antibody were from Cell Signaling Technology, Beverly, MA, USA. Dexamethasone (DEX) was from Hubei Tianyao Pharmaceutical Co., Ltd., Xiangyang, China. HPLC-grade methanol, acetonitrile, and formic acid were purchased from Fisher Chemicals Co., NJ, USA. Ultrapure water was freshly prepared with the Millipore water purification system, MA, USA.

### 2.2. High-Performance Liquid Chromatography Coupled with Quadrupole Time of Flight Mass Spectrometry (HPLC–QTOF–MS) Analysis of EEIH

EEIH was prepared as previously described [18]. An aliquot of EEIH was dissolved in methanol at the concentration of 0.5 mg/mL. The solution was filtered using a 0.45–μm Millipore filter for HPLC–QTOF–MS analysis [24].

HPLC–QTOF–MS analysis was performed on Agilent series 1290 Infinity HPLC instrument coupled with an Agilent 6530 Q-TOF mass spectrometer (Agilent Technologies, Santa Clara, CA, USA) equipped with an electrospray ionization interface. The HPLC instrument included a binary pump, an autosampler, an online degasser, and a thermostatically controlled column compartment. A ODS-C18 column (150 mm × 2.0 mm I.D., particle size 5 μm) column was used. The mobile phase consisted of 0.1% formic acid (A) and acetonitrile (B). The linear gradient was as follows: 0–20 min, 5–10% B, 20–20.1 min, 10–90% B, 20.1–25 min, 90% B, 25.0–25.1 min, 90–5% B, 25.1–30 min, 5% B. The whole analysis took 30 min. The flow rate was 0.3 mL/min. The column temperature was set at 30 °C.

Mass spectrometric detection was operated in positive electrospray ionization mode. The operating parameters were as follows: capillary voltage, 4000 V, nebulizer pressure, 40 psi, drying gas flow rate, 9 l/min, gas temperature, 350 °C, skimmer voltage, 60 V, octapole rf, 250 V, fragmentor voltage, 175 V. Mass spectra were acquired in the range of 50–1000 *m/z*. The instrument performed the internal mass calibration automatically by an automated calibrate delivery system. The calibrating solution contained the internal reference masses at *m/z* 121.0508 and 922.0098. All the data acquisition was controlled by Agilent Mass Hunter software (version B.01.03 Build 1.3.157.0 2).

### 2.3. In Vitro Antioxidant Activity of EEIH

The DPPH and ABTS radical scavenging activity and ferric reducing antioxidant power (FRAP) of EEIH were determined as reported previously [25]. Vitamin C was used as the antioxidant reference standard and the results were expressed as vitamin C equivalents (mg VCE/g).

### 2.4. Experimental Animals and Treatment

**Figure 1:**
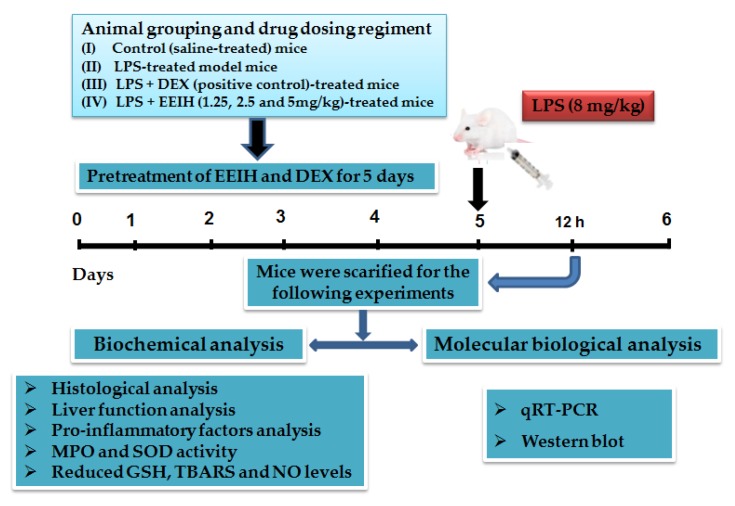
Experimental protocol for lipopolysaccharide (LPS)-induced acute liver injury (ALI) model and treatment processes.

Male BALB/c mice aged 5–6 weeks were purchased from Shanghai Experimental Animal Center of Chinese Academy of Sciences, Shanghai, China (certificate no. SCXK 2007-0005). Mice were acclimatized for one week prior to use. The protocol of the mice experiment was presented in Figure 1. LPS-induced liver injury model mice were established as described previously [26]. The doses of EEIH were selected based on the pre-experiment. Dexamethasone (DEX, 1.8 mg/kg, *i.p.*) was used as a positive control [4]. Before establishing the ALI model, mice were randomly divided into six groups (*n* = 5):

(I) Normal control group: mice were injected intraperitoneally (*i.p.*) with saline every day for six days.

(II) Model control group: mice were injected *i.p.* with saline every day for five days and then injected *i.p.* with LPS (8 mg/kg).

(III) LPS + DEX (positive control) group: mice were injected *i.p.* with DEX at the dose of 1.8 mg/kg every day for five days, and then injected *i.p*. with LPS (8 mg/kg).

(IV) LPS + EEIH (1.25, 2.5, or 5.0 mg/kg) group: mice were injected *i.p.* with EEIH at the doses of 1.25, 2.5, or 5.0 mg/kg every day for five days, and then injected *i.p*. with LPS (8 mg/kg).

The dose-volume was 0.2 mL/10 g body weight. One hour after the last administration, the mice were administered *i.p*. with LPS (8 mg/kg). Mice were anesthetized with 10% chloral hydrate and then sacrificed at 12 h after LPS injection. The blood samples and liver tissues were collected for analysis. The liver index was calculated as following equation:

Liver index = liver weight/mouse body weight × 100 (g/100 g body weight).

This study was carried out in accordance with the guidelines established by the Institute for Experimental Animals of Zhejiang University and the protocol was approved by the University Local Committee on the Ethical Use of Animal experiments (ethical code: 14878).

### 2.5. Biochemical Examinations of Serum ALT and AST

The serum alanine aminotransferase (ALT) and aspartate aminotransferase (AST) levels were measured using the Roche Cobas C311 Chemistry Analyzer (Roche Diagnostics, Indianapolis, IN, USA). The values were expressed as U/L of serum.

### 2.6. Liver Histopathology Observation

The collected liver tissues were fixed with 4% paraformaldehyde, and then were dehydrated through a series of graded ethanol, hyalinized with xylene, embedded in paraffin, and sectioned at 5 μm thicknesses. Microsections were stained with hematoxylin and eosin (H&E). The histological changes were observed on an Olympus CKX53 microscope at a fixed 100× magnification.

### 2.7. Measurement of Cytokines

Liver tissue samples (ca. 70 mg) were washed in pre-cooled saline to eliminate extra blood and then they were mixed with 630 μL of PBS to make 10% homogenate through a tissue homogenizer. The homogenate supernatant was collected by centrifugation at 12,000 rpm for 10 min at 4 °C. The protein concentrations in the homogenate supernatants were detected by BCA method using bovine serum albumin as a standard. Then, the concentrations of IL-6, IL-1β, and TNF-α in the supernatants were detected using commercial ELISA kits as previously described [27]. The values were expressed as pg/mg protein based on appropriate standard curve.

### 2.8. Measurement of MPO and SOD Activities and MDA, NO and Reduced GSH Levels in Liver Tissue

The activities of MPO and SOD in homogenate and the level of reduced GSH in the supernatants were measured by spectrophotometry using commercial diagnostic kits. The hepatic MPO activities were standardized per gram wet tissue, while SOD activities and reduced GSH levels were standardized to the protein in each sample. Lipid peroxidation was measured by thiobarbituric acid reactive substances (TBARS) of mainly malondialdehyde (MDA) [28]. Liver NO levels were measured using Griess reagents. The levels of MDA and NO in liver tissue homogenate supernatants were determined using commercial assay.

### 2.9. Quantitative Real-Time PCR (qRT-PCR)

The total RNA was isolated from liver tissue using TRIzol reagent and reverse transcription was performed as previously described [18]. The amplification was performed on the Bio-Rad CFX96 real-time PCR system in a 20 μL reaction system using FastStart Universal SYBR Green Master. The qPCR cycling was performed as follows: initial denaturation at 95 °C for 10 min, followed by 40 cycles of denaturation at 95°C for 10s, and then annealing at 60 °C for 1 min. The primers for qRT-PCR were synthesized by Sangon Biotech (Shanghai) Co. Ltd., Shanghai, China, and the sequences are listed in Table S1. GAPDH was used as an endogenous control. The mRNA expression levels of the tested genes relative to GAPDH were determined using the 2^−ΔΔCt^ method and shown as fold induction.

### 2.10. Western Blotting

Liver tissues were homogenized with RIPA lysis buffer supplemented with protease and phosphatase inhibitors (cocktail) and then centrifuged at 12,000 rpm for 15min at 4 °C. The protein contents were detected using the BCA protein assay kit. The denatured proteins were separated on 10% SDS-PAGE and then transferred to a polyvinylidene fluoride membrane (0.45 μM). After blocking the membrane with 5% skim milk in Tris-buffered saline containing 0.1% Tween-20 (TTBS) for 2 h at 37 °C, the blot was incubated with anti-mouse actin monoclonal antibody, anti-rabbit TLR4, NF-κB p65, or phospho-NF-κB p65 polyclonal antibody in TTBS containing 5% skim milk overnight at 4 °C. The membranes were washed with TTBS and then incubated with HRP-conjugated goat anti-mouse or anti-rabbit IgG (H+L) for 2 h. After washing the membrane with TTBS three times, the signal was visualized with BeyoECL Star kit using the LiCor C-DiGit Blot Scanner (LiCor, Lincoln, NE, USA).

### 2.11. Statistical Analysis

The normality of the distribution of each variable was measured through means of the Kolmogorov–Smirnov test. The data were expressed as mean ± standard deviation (SD) and examined for their statistical significance of difference with ANOVA and a Tukey *post-hoc* test. The calculations and graphs were produced using Prism 7 software (GraphPad Software, USA). *P*-values of less than 0.05 were considered to be statistically significant.

## 3. Results

### 3.1. The Annotation and Structural Characterization of the Compounds in EEIH

**Figure 2:**
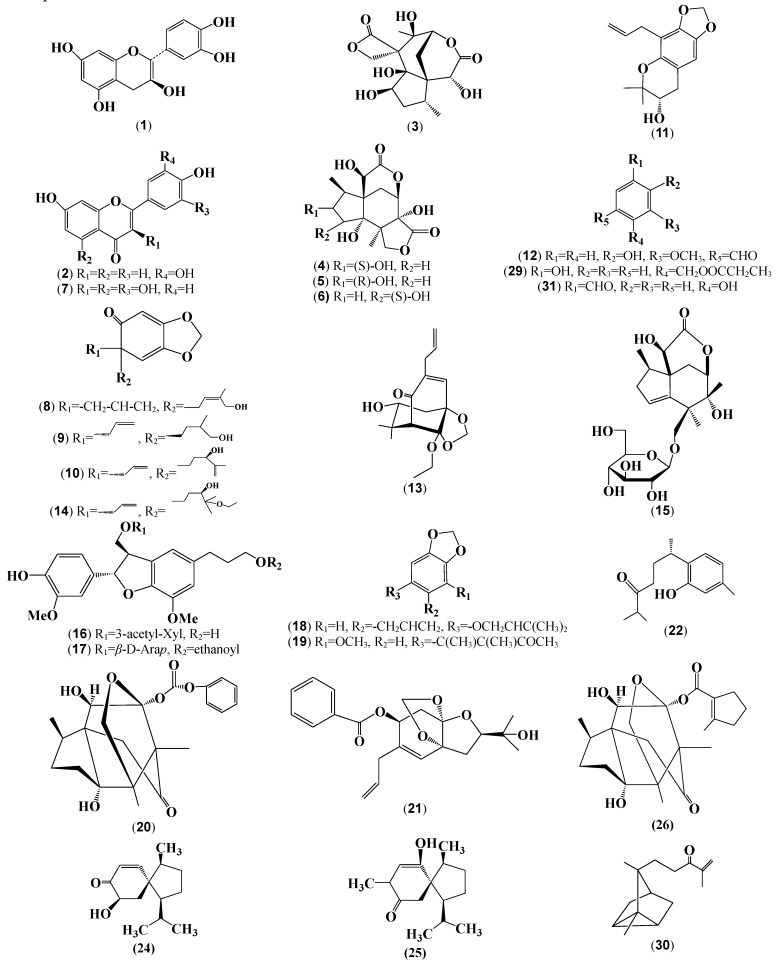

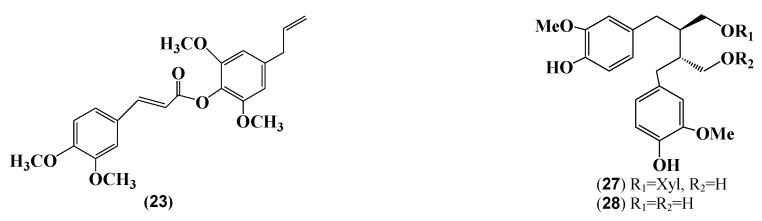
The chemical structures of the annotated compounds in EEIH.

The total ion chromatogram of EEIH in positive ion mode was shown in Figure S1. The MS/MS data were extracted from the raw data and manually interpreted based on the previous research [19,20,21,22,23,29,30,31,32,33,34,35,36,37]. A total of 24 peaks in EEIH were attributed to 31 compounds due to the presence of isomer. These compounds mainly included flavonoids such as (+)-catechin (**1**), 3′,4′,7-trihydroxyflavone (**2**), and taxifolin (**7**), sesquiterpenoids such as anisatin (**3**), (2S)-hydroxyneomajucin (**4**), (2R)-2-hydroxyneomajucin (**5**), majucin (**6**), henrylactone D (**15**), tashironin (**20**), 1*β*-isopropyl-4-*β*-methyl-9*β*-hydroxy spiro [4,5] dec-6-en-8-one (**24**), 10-hydroxyacoronene (**25**), and tashironin A (**26**), ligans such as dihydrodehydrodiconiferyl alcohol 9-*O*-*β*-D-(3′-*O*-acetyl)-xylopyranoside (**16**), illiciumlignan F (**17**), 4-allyl-2,6-dimethoxy- phenyl-3,4-di-methoxycinnamate (**23**), (-)secoisolariciresinol-*O*-*α*-L-rhamnopyranoside (**27**), and (+)-isolariciresinol (**28**), as well as simple phenylpropanoids such as illihenryione E (**8**), illihenryione C (**9**), illifrognone D (**10**), burmanicumol D (**11**), illioliganone K (**13**), illifrognone E (**14**), 1-allyl-2-(3-methylbut-2-enyloxy)-4,5-methylenedioxybenzene (**18**), trans-3-methoxy-4,5-methy- lene-dioxycinnamaldehyde (**19**), illioliganfuranol A (**21**), and (-)-1-hydroxy-1,3,5-bisabola- trien-10-one (**22**). The other compounds including vanillin (**12**), 2-(4-hydroxyphenyl) ethyl acetate (**29**), α-santal-11-en-10-one (**30**), and hydroxybenzaldehyde (**31**) were found in the EEIH. The quasi-molecular ion, MS/MS fragments ions of these annotated compounds in EEIH were listed in Table 1 and their chemical structures were shown in Figure 2.

### 3.2. In Vitro Antioxidant Activities of EEIH

The DPPH, ABTS, and FRAP assays were usually used to evaluate the in vitro antioxidant capabilities of plant extracts [38]. The antioxidant activities of EEIH were 22.00 ± 1.26, 141.55 ± 7.39, and 10.05 ± 0.07 mg VCE/g for DPPH, ABTS, and FRAP assays, respectively. The results suggested that EEIH possessed in vitro potent antioxidant activities.

### 3.3. Effect of EEIH on Liver Index, Serum ALT and AST Levels in LPS-Induced ALI Mice

**Figure 3:**
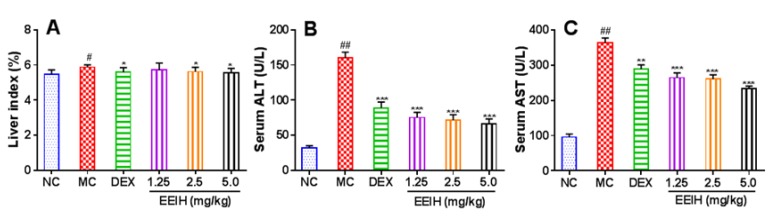
Effects of EEIH on the liver index (**A**), serum alanine aminotransferase (ALT, **B**) and aspartate aminotransferase (AST, **C**) levels in LPS-induced acute liver injury mice. The values are presented as the means ± SD (*n* = 5). *P* values were determined by ANOVA and a Tukey *post-hoc* test. Significant differences compared to the normal control group (NC) are designated as ^#^ *p* < 0.01 and ^##^ *p* < 0.001, those compared to the model control group (MC) as ^*^ *p* < 0.05, ^**^ *p* < 0.01, and ^***^ *p* < 0.001. DEX: dexamethasone (positive control).

Liver disorders generally accompanied the increase of the hepatic index. As shown in Figure 3A, there were significant increases in the liver index of the LPS-treated mice compared with the normal control group (*p* < 0.05). The pretreatment with DEX and EEIH at 2.5 and 5 mg/kg significantly diminished the liver index in LPS-treated mice (*p* < 0.05), indicating that EEIH could prevent liver injury in mice induced by LPS. Serum AST and ALT have been commonly used as biochemical markers for evaluating liver functions in the experimental and clinical liver injury [26]. Effect of EEIH on the serum ALT and AST levels in LPS-treated mice was shown in Figure 3B,C. The serum ALT and AST levels were significantly elevated in mice induced by LPS (*p  *<  0.001). The pretreatment with EEIH and DEX markedly decreased the serum AST and ALT levels in LPS-treated mice (*p* < 0.01 or *p* < 0.001). These results suggested that EEIH could prevent LPS-induce ALI and improve liver functions in mice.

### 3.4. Effects of EEIH on Liver Histopathology and MPO Activity in LPS-Induced ALI Mice

**Figure 4:**
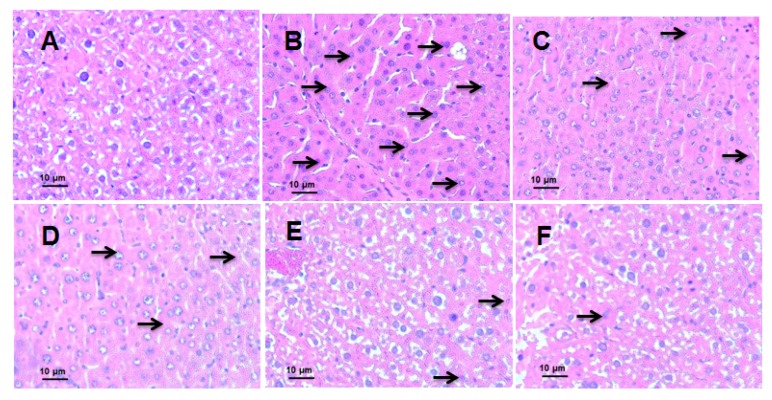

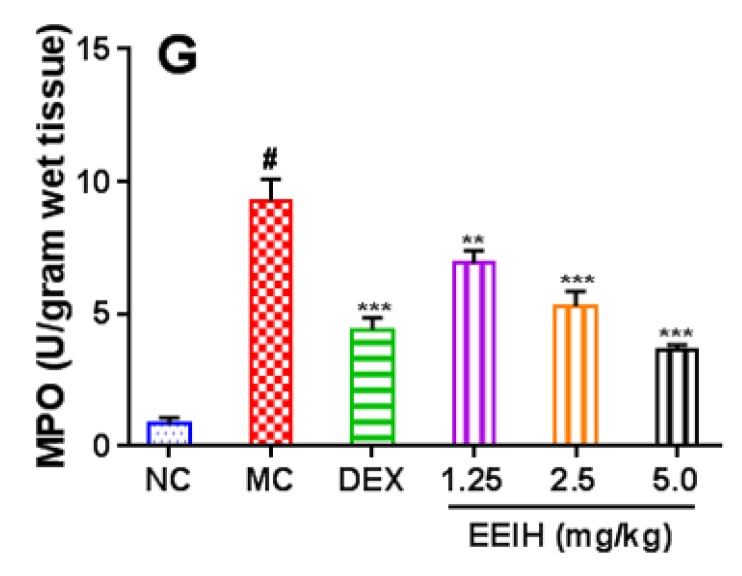
Effects of EEIH on histopathological changes (**A**–**F**) and MPO activity (**G**) of liver tissue in LPS-induced ALI mice. The liver sections were stained using H&E. The light photomicrographs shown were representative of liver sections from five mice per group. The black arrows showed inflammatory cells in liver tissues. (**A**) Normal control (NC), (**B**) model control (MC), (**C**) dexamethasone (DEX, positive control), (**D**) EEIH (1.25 mg/kg), (**E**) EEIH (2.5 mg/kg), and (**F**) EEIH (5.0 mg/kg). The values are presented as the means ± SD (*n* = 5). *P* values were determined by ANOVA and a Tukey *post-hoc* test. Significant differences compared to the NC group are designated as ^#^ *p* < 0.001; those compared to MC group as ^**^ *p* < 0.01 and ^***^ *p* < 0.001.

Effects of EEIH on the liver histopathology in LPS-treated mice were shown in Figure 4A–F. The liver tissue in the normal control group displayed normal hepatocytic architecture and a visible nucleus (Figure 4A). Conversely, a remarkable liver pathological changes such as vacuolization, hemorrhage, hepatocyte necrosis, inflammatory infiltration, and liver lobule destruction was observed in model control group (Figure 4B), suggesting successful LPS-induced ALI model in this study. The pretreatment with EEIH (1.25, 2.5, and 5 mg/kg) and DEX significantly improved the histopathological changes in LPS-induced ALI mice (Figure 4C–F). The aggregation of neutrophils in liver tissue reflects the degree of inflammation. As shown in Figure 4G, LPS significantly enhanced the activities of the neutrophil marker MPO in mouse liver tissue compared with normal control group (*p* < 0.001). However, the pretreatment of EEIH and DEX significantly dose-dependently decreased the MPO activities in the liver tissue of LPS-treated mice (*p* < 0.001), suggesting that EEIH could protect mice against ALI induced by LPS.

### 3.5. Effects of EEIH on the mRNA and Protein Expression Levels of Proinflammatory Factors in Liver Tissue of LPS-Induced ALI Mice

**Figure 5:**
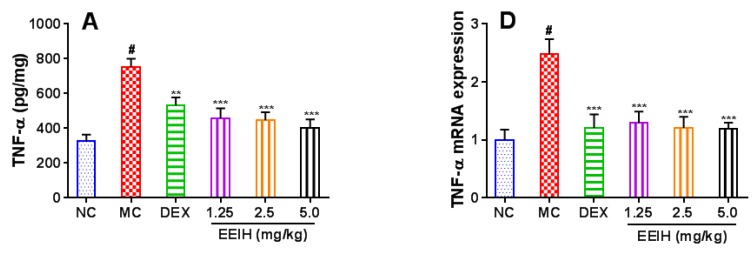

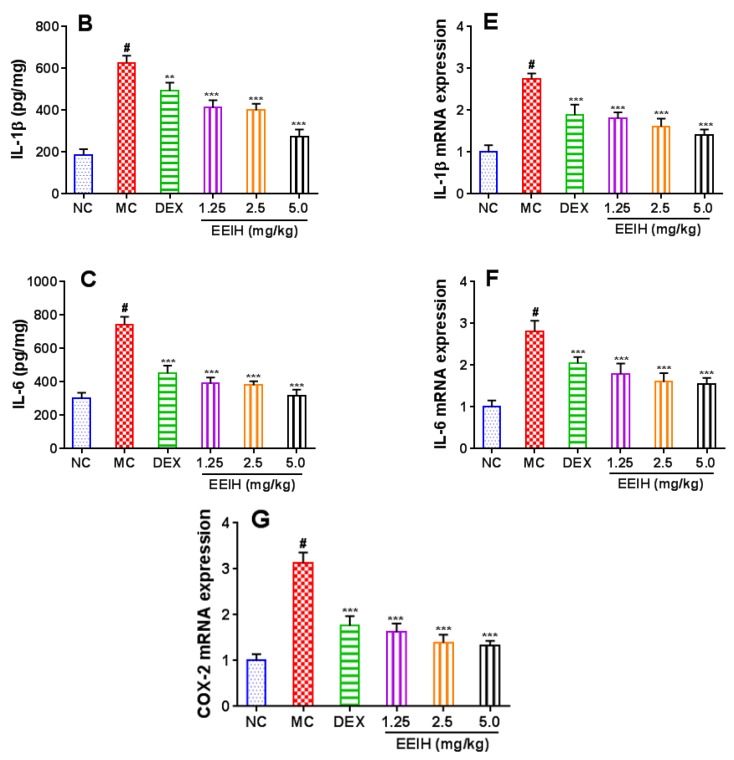
Effects of EEIH on the mRNA and protein expression levels of pro-inflammatory factors in liver tissue of LPS-induced ALI mice. (**A**–**C**) The levels of TNF-α, IL-1β, and IL-6 in liver tissue were measured using ELISA. (**D**–**G**) The mRNA expression levels of TNF-α, IL-1β, IL-6, and COX-2 in liver tissue were measured using qRT-PCR. The values are presented as the means ± SD (*n* = 5). *P* values were determined by ANOVA and a Tukey *post-hoc* test. Significant differences compared to the normal control group (NC) are designated as ^#^ *p* < 0.001, those compared to model control group (MC) as ^**^ *p* < 0.01, and ^***^ *p* < 0.001.

The levels of proinflammatory cytokines in liver tissue are correlative with the inflammatory severity of ALI in mice [39]. The levels of proinflammatory cytokines TNF-α, IL-1β, and IL-6 in liver tissue were first measured by ELISA, and the results were shown in Figure 5A–C. LPS led to a significant increase in the levels of TNF-α, IL-1β, and IL-6 in mouse liver tissue compared to the normal control group (*p* < 0.001). The administration of EEIH at three doses and DEX significantly decreased the levels of TNF-α, IL-1β, and IL-6 in liver tissue of LPS-induced ALI mice (*p* < 0.001). Furthermore, the mRNA expression levels of the proinflammatory cytokines were further determined by qRT-PCR. As shown in Figure 5D–F, the pretreatment with EEIH at three doses and DEX significantly suppressed the upregulated mRNA expression of TNF-α, IL-1β, and IL-6 in liver tissue of LPS-treated mice (*p*  <  0.001). Similarly, LPS also markedly upregulated the expression levels of COX-2 mRNA in mice, while the pretreatment with EEIH and DEX resulted in remarkable inhibition in the upregulated COX-2 mRNA expression levels in liver tissue of LPS-treated mice (*p*  <  0.001, Figure 5G). These findings further confirmed the anti-inflammatory mechanism of protective activity of EEIH against LPS-induced ALI in mice.

### 3.6. Effects of EEIH on the mRNA and Protein Expression Levels of TLR4 and NF-κB in Liver Tissue of LPS-Treated Mice

**Figure 6:**
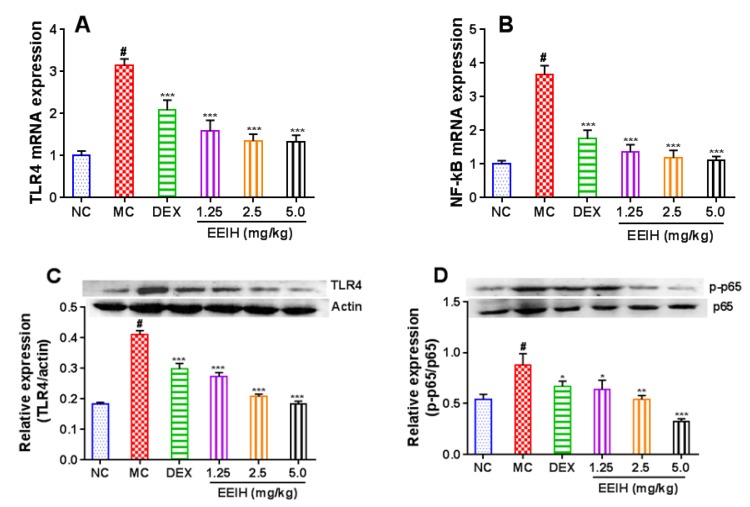
Effects of EEIH on the mRNA (**A**,**B**) and protein (**C**,**E**) expression levels of toll-like receptor 4 (TLR4) and nuclear factor-κB (NF-κB) p65 in liver tissue of LPS-treated mice. The figures shown were representative of five mice per group. The values are presented as the means ± SD (*n* = 5). *P* values were determined by ANOVA and a Tukey *post-hoc* test. Significant differences compared to the normal control group (NC) are designated as ^#^ *p* < 0.001, those compared to the model control group (MC) as ^*^ *p* < 0.05, ^**^ *p* < 0.01, and ^***^ *p* < 0.001. DEX: dexamethasone (positive control).

LPS induces the immune response through TLR4, which further activate NF-κB [40]. LPS induces ALI via initiation of TLR4/NF-κB pathway following by production of pro-inflammatory cytokines and enzyme [12]. To investigate the inhibitory mechanism of EEIH against inflammatory response, the mRNA expression levels of TLR4 and NF-κB in liver tissue were first measured by qRT-PCR, and the results were shown in Figure 6A,B. The mRNA expression levels of TLR4 and NF-κB were dramatically upregulated in liver tissue of LPS-treated mice compared to the normal control group (*p* < 0.001). The administration of EEIH and DEX significantly downregulated the mRNA expression levels of TLR4 and NF-κB p65 in liver tissue of LPS-induced ALI mice (*p* < 0.001). The protein expression of TLR4 and the phosphorylation of NF-κB p65 in the liver tissue of LPS-treated mice were also examined using Western blot. LPS notably promoted the protein expression of TLR4 and the phosphorylation of NF-κB p65 (Figure 6C,D). Pretreatment with EEIH at three doses and DEX remarkably inhibited the protein expression of TLR4 and the phosphorylation of NF-κB p65 in the liver tissue of LPS-induced ALI mice. These results suggested the role of anti-inflammatory activity of EEIH by inhibiting LPS-activated TLR4/NF-κB pathway behind the observed hepatoprotective activity.

### 3.7. Effects of EEIH on Oxidative and Nitrosative Stress and the mRNA Expression Levels of Nrf2 in Liver Tissue of LPS-Treated Mice

**Figure 7:**
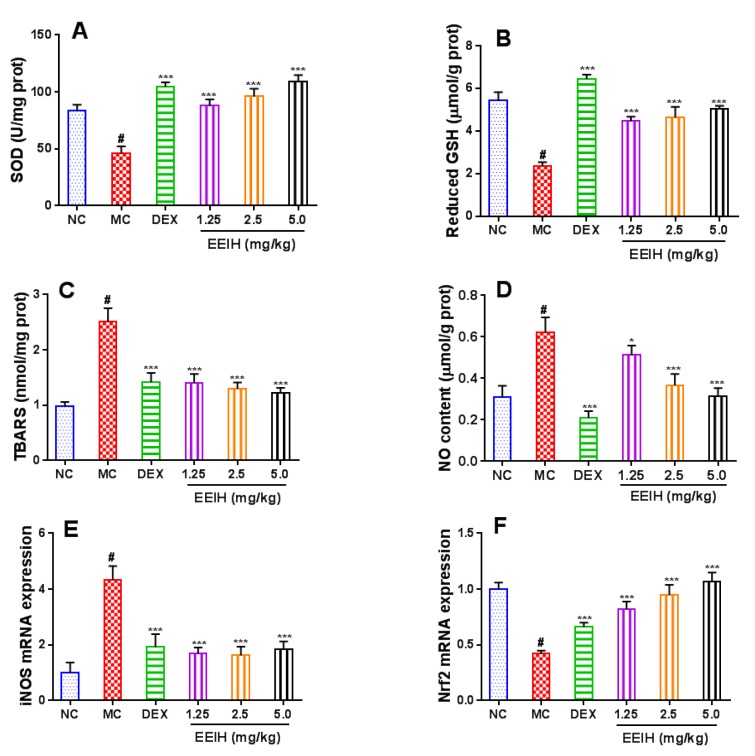
Effects of EEIH on SOD (**A**), reduced GSH (**B**), TBARS (**C**), and NO (**D**) levels as well as the mRNA expression of iNOS (**E**) and Nrf2 (**F**) in liver tissue of LPS-treated mice. The values are presented as the means ± SD (*n* = 5). *P* values were determined by ANOVA and a Tukey *post-hoc* test. Significant differences compared to the normal control group (NC) are designated as ^#^ *p* < 0.001, those compared to model control group (MC) as ^*^ *p* < 0.05 and ^***^ *p* < 0.001.

Oxidative and nitrosative stress play an important role in the occurrence and development of ALI. As shown in Figure 7A–C, LPS significantly decreased the SOD activity and the level of reduced GSH, and increased TBARS level in mouse liver tissue compared with normal control mice (*p* < 0.001). The pretreatment with EEIH and DEX significantly enhanced the SOD activity and the level of reduced GSH as well as decreased TBARS levels in liver tissue of the LPS-induced ALI mice (*p* < 0.001). LPS also significantly induced the production of NO (Figure 7D) and the mRNA expression of iNOS (Figure 7E) in mouse liver tissue (*p* < 0.001). EEIH pretreatment markedly inhibited the production of NO and the mRNA expression of iNOS in liver tissue of the LPS-treated mice (*p* < 0.05 or *p* < 0.001), suggesting that EEIH significantly alleviated nitrosative stress. Nrf2, a redox-sensitive transcriptional factor, plays a pivotal role in regulating the oxidative stress and cytokines expression in ALI mice [14]. Therefore, the mRNA expression levels of Nrf2 in liver tissue were further determined using qRT-PCR, and the results were shown in Figure 7F. The lower mRNA expression level of Nrf2 was detected in the liver tissue of LPS-induced ALI mice compared with normal control mice (*p* < 0.001). The pretreatment of EEIH significantly dose-independently upregulated the mRNA expression levels of Nrf2 in liver tissues in LPS-induced ALI mice close to the levels of normal control mice (*p* < 0.001), indicating that the antioxidant properties of EEIH were be attributed to Nrf2 activation. These results suggested that EEIH alleviated hepatic oxidative and nitrosative stress burden behind its observed hepatoprotective activity.

## 4. Discussion

In our previous study, EEIH has been demonstrated to be rich in polyphenols with a significant amount of total phenolic and flavonoid content [18]. In this study, in vitro analysis showed that EEIH possessed potent antioxidant activities. To elucidate potential compounds responsible for its antioxidant activity, EEIH was subjected to HPLC–QTOF–MS analysis. EEIH mainly contained flavonoids, sesquiterpenoids, ligans, simple phenylpropanoids, and other compounds (Table 1 and Figure 2). Flavonoids from natural plants have been showed to have ROS-scavenging, anti-inflammatory, and hepatoprotective effects indicative of its potentiality as a therapeutic agent for ALI [41,42]. Catechin (**1**) was reported to have superior antioxidative abilities compared to antioxidant nutrients vitamin C, *β*-carotene, and vitamin E [43] and scavenge free radicals through decreasing the MDA content and enhancing the activity of SOD [44]. Taxifolin (**7**), also named as dihydroquercetin, was reported to possess potent antioxidative activities and has been widely used in the food and healthcare industries as food additives [45,46]. It could ameliorate concanavalin A-induced mouse experimental fulminant hepatitis [47], and defense tetrachloromethane hepatitis in rats [48] through Nrf2-dependent antioxidant pathway. Catechin (**1**) and taxifolin (**7**) protected against rotenone-induced kidney toxicity in rats via alleviating oxidative stress [49]. 3′,4′,7-trihydroxyflavone (**2**) ameliorated H_2_O_2_-induced cell death through decreasing the intracellular ROS generation and recovery of antioxidant enzymes [50]. The antioxidant activity of EEIH might be attributed to the flavonoid compounds such as catechin (**1**), 3′,4′,7-trihydroxyflavone (**2**), and taxifolin (**7**), which are known for their antioxidant potential.

DEX has been reported to possess hepatoprotective effect against LPS-induced liver injury by down-regulating glucocorticoid-induced tumor necrosis factor receptor ligand [51] and through inhibition of inflammatory and oxidative stress [52]. DEX has been also used to treat endotoxic shock in clinical studies [53,54] and selected as a positive control for the hepatoprotective effect against LPS-induced liver injury [4].

ALI is a life-threatening disease with high morbidity and mortality rates. Endotoxin-induced hepatotoxicity remains a major source of death [55]. The liver index was usually used to assess liver damage in animal experiments [56]. In this study, LPS significantly increased the liver index and EEIH pretreatment significantly decreased this index in LPS-treated ALI mice.

ALT and AST are the preferred indicators for the evaluation of liver injury [57]. During liver injury, these enzymes enter into the bloodstream resulting in enhanced serum ALT and AST levels [58]. The serum ALT and AST levels were positively correlated with liver histopathology [59]. EEIH significantly decreased the elevated serum ALT and AST levels in LPS-treated mice. The H&E staining results revealed that EEIH pretreatment preserved the hepatic cell architecture and decreased inflammatory infiltration. Neutrophil infiltration is an initial reaction of liver injury. The neutrophil aggregation in liver tissue reflects the degree of inflammation [60]. Our results demonstrated that EEIH pretreatment decreased the MPO activity in liver tissue of LPS-induced ALI mice. These results suggested that EEIH protected the liver from injury in mice induced by LPS.

The excessive production of proinflammatory cytokines is a hallmark of the pathological process of ALI [61]. The levels of proinflammatory cytokines TNF-α, IL-1β, and IL-6 were enhanced in LPS-induced ALI mice [62]. The effects of EEIH on proinflammatory cytokines were investigated. The results revealed that EEIH pretreatment notably inhibited the production and mRNA expression of TNF-α, IL-1β, and IL-6 in liver tissue of LPS-induced ALI mice. Similarly, COX-2 is an essential inflammatory mediator that plays a crucial role in the pathological process of inflammation [63]. Due to hepatic injury, Kupffer cells are activated leading to elevation of COX-2 expression, which in turn aggravates hepatic inflammation [64]. The pretreatment with EEIH down-regulated the mRNA expression levels of COX-2 in liver tissue of LPS-treated mice. These investigations demonstrate that EEIH might alleviate liver injury through its anti-inflammatory activities.

LPS was reported to triggers the hepatic Kupffer cells to secrete inflammatory cytokines via TLR4 [65]. TLR4 played a significant role in liver dysfunction induced by inflammation [4]. An increased level of TLR4 was found in septic patients [66] and TLR4 was excessively expressed in LPS-induced ALI [4]. EEIH significantly downregulated the mRNA and protein expression levels of TLR4 in the liver tissue from LPS-induced ALI mice, which was consistent with the previous report [4]. Stimulation of TLR4 by LPS could activate NF-κB in the liver to induce the mRNAs expression and production of inflammatory cytokines [12]. It was reported that a substantial decrease in hepatic NF-κB activation has been reported in LPS-induced fulminant hepatic failure TLR4-deficient mice [67]. EEIH significantly down-regulated the mRNA expression level and inhibited the phosphorylation of NF-κB p65 in liver tissue of LPS-treated mice. These results indicated that EEIH might alleviate hepatic inflammation via blocking TLR4 and NF-κB activation and subsequently inhibiting the production of pro-inflammatory cytokines. This partly explained why EEIH treatment could mitigate LPS-induced hepatic damage.

ROS have been reported to induce the activation of NF-κB and the expression of iNOS. When sepsis happens, iNOS is secreted from vascular endothelial cells to accelerate the production of high nitric oxide (NO) [68]. The excessive accumulation of NO contributes to the formation of extra toxic peroxynitrite after response with superoxide anions [69], which ultimately results in nitrosative stress [70]. LPS induced a substantial increase in hepatocyte nitric oxide contributing to nitrosative stress and the subsequent liver toxicity [71]. In the present study, EEIH significantly inhibited the production of NO and downregulated the mRNA expression levels of iNOS in liver tissues of LPS-induced ALI mice. Our findings suggested that EEIH possessed the protective activity against liver nitrosative stresses and consequently ameliorates liver dysfunction.

Given the role of ROS in NF-κB activation and the consequent secrete of pro-inflammatory cytokines, oxidative stress plays a significant role in the pathogenesis of LPS-induced ALI [72]. LPS-exposure bursts ROS production and inhibits the antioxidant defense process, which is responsible for scavenging excessive ROS formation, contributing to oxidative damage in the hepatocyte [73]. Furthermore, oxidative stress and consequent free radicals generation in liver tissue can exacerbate inflammation, resulting in liver injury. Enzymatic and non-enzymatic antioxidant in the body might scavenge the free radicals to protect the cells from oxidative injury. SOD is the primary defense to eradicate ROS and is efficient in preventing and treating liver damage associated with superoxide free radicals. When superoxide anion radicals are extremely generated or the SOD concentration is low, superoxide anions will induce oxidation [56]. SOD can catalyze the reactive superoxide anion O_2_^−^ into O_2_ and hydrogen peroxide (H_2_O_2_) [74]. GSH, the primary non-enzymatic antioxidant, protects the cells from oxidative injury through efficiently eradicating lipids and other organic peroxides [75]. In this study, LPS significantly reduced the liver SOD and GSH levels, while EEIH could restore the levels of SOD and reduced GSH in LPS-treated mice, suggesting its antioxidant defense function against LPS-induced ALI. MDA is a final product of lipid peroxidation, which is considered as an indicator of ROS for measuring the degree of oxidative stress [76]. LPS increased the ROS levels, which accelerate lipid peroxidation-mediated cytotoxic MDA formation that culminates to oxidative liver damage [13]. EEIH pretreatment significantly decreased the MDA levels in liver tissue of LPS-induced ALI mice. These findings suggest that EEIH could provide liver protection against LPS-induced oxidative damage by suppressing lipid peroxidation and promoting the activities and expression of antioxidant enzymes. These results were positively correlated well with those of its in vitro antioxidant experiments. In our previous study, EEIH has also been proved to inhibit LPS-induced ROS production in RAW246.7 cells [24].

Nrf2 is primarily expressed in metabolic and detoxifying tissue of the liver and protects cells from oxidative stress [77]. Under oxidative stimuli, Nrf2 transfers from the cytoplasm to the nucleus where it binds to the antioxidant response element (ARE) site to induce the expression of endogenous detoxifying and antioxidant enzymes such as SOD, CAT, and GSH-Px [78]. These enzymes play significant roles in ROS elimination and GSH synthesis that contribute to stresse alleviation [79]. It was reported that the activation of Nrf2 protected against LPS-induced ALI [14,15]. In this study, a remarkable downregulation of Nrf2 mRNA expression level in LPS-treated mice, which were consistent with previous reports [80,81]. EEIH pretreatment, however, upregulated the mRNA expression level of Nrf2 in liver tissues of LPS-induced ALI mice to normal levels. Therefore, EEIH could suppress liver oxidative stress damage by activating Nrf2 pathway. Meanwhile, the anti-inflammatory activity of EEIH might also be related to its capacity to upregulate the mRNA expression of Nrf2. It was reported that Nrf2 inhibited the activation of NF-κB and the production of NO and pro-inflammatory cytokines in LPS-induced ALI [14].

## 5. Conclusions

**Figure 8:**
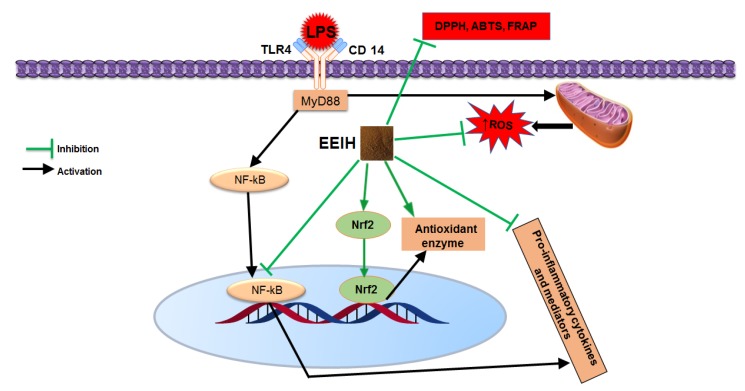
A proposed schematic diagram illustrating the mechanism of protection of EEIH against LPS-induced acute liver injury. EEIH: ethanol extract of *Illicium henry*, LPS: lipopolysaccharide, DPPH: 2,2-diphenyl-1-picrylhydrazil, ABTS: 2,2′-azino-bis-(3-ethylbenzothiozoline-6-sulfonic acid) disodium salt, FRAP: ferric-reducing antioxidant power, ROS: reactive oxygen species, TLR4: toll-like receptor 4, NF-κB: nuclear factor-κB, Nrf2: nuclear factor erythroid 2-related factor 2.

In summary, this investigation demonstrated that EEIH possessed protective effects against LPS-induced ALI in mice via alleviating inflammatory response, oxidative and nitrosative stress burden through activating the Nrf2 and suppressing the TLR4/NF-κB signaling pathways (Figure 8). EEIH might be favorable for the treatment of endotoxin-induced liver damages and dysfunction and act as a potential hepatoprotective agent. The hepatoprotective activity of EEIH might be associated with the flavonoid compounds such as catechin (**1**), 3′,4′,7-trihydroxyflavone (**2**), and taxifolin (**7**) that most possibly act synergistically. This study will benefit the development and utilization of the root bark of *I. henryi* and provide reliable evidence for its future clinical applications in modern medicine.

## Figures and Tables

**Table 1:** MS data and annotated compounds in ethanol extract of *I. henryi* (EEIH) by HPLC–QTOF–MS/MS.

Peaks	Rt (min)	Molecular Formula	QMI (*m/z*)	MS/MS Fragment Ions (Relative Abundance)	Annotated Compound	Ref.
1	3.493	C_15_H_14_O_6_	[M + H]^+^ (291.0867)	123.0431 (51.72), 139.0372 (100.00), 161.0607 (9.04), 207.0630 (10.39), 231.1076 (1.18), 258.1289 (0.76), 291.0867 (0.83)	(+)-Catechin (**1**)	[20]
2	5.127	C_15_H_10_O_5_	[M + H]^+^ (271.0541)	107.0439 (21.19), 137.0542 (100.00), 173.0532 (16.42), 197.0507 (13.28), 227.1040 (7.83), 271.0541 (9.38)	3′,4′,7-Trihydroxyflavone (**2**)	[29]
3	5.536	C_15_H_20_O_8_	[M + NH_4_]^+^ (346.1505)	107.0464 (94.61), 147.0432 (100.00), 195.0804 (65.03), 222.0607 (85.62), 301.1067 (21.00), 346.1505 (9.21)	Anisatin (**3**), (2S)-Hydroxy- neomajucin (**4**), (2R)-2-hy- droxyneomajucin (**5**), majucin (**6**)	[19]
4	6.288	C_15_H_12_O_7_	[M + H]^+^ (305.0667)	111.0432 (4.00), 123.0432 (66.39), 153.0164 (100.00), 195.0260 (12.47), 231.0626 (87.29), 259.0575 (31.005), 287.0535 (31.05), 305.0667 (8.02)	Taxifolin (**7**)	[30]
5	6.550	C_15_H_18_O_4_	[M + H]^+^ (263.1285)	105.0665(29.06), 135.0775 (100.00), 159.1145 (47.49), 185.0860 (24.58), 203.1038(37.05), 227.1041 (18.19), 263.1285 (9.02)	Illihenryiones E (**8**), C (**9**), illifrognone D (**10**), burmanicumol D (**11**)	[23,31]
6	7.155	C_8_H_8_O_3_	[M + H]^+^ (153.0609)	107.0800 (100.00), 119.0787 (6.92), 135.1107 (26.23), 142.0674 (7.76), 153.0609 (15.98)	Vanillin (**12**)	[32]
7	7.285	C_17_H_24_O_5_	[M + Na]^+^ (331.1550)	119.0488 (13.31), 151.0727 (100.00), 189.0903 (32.84), 227.1042 (43.28), 255.0985 (31.55), 285.1108 (19.79), 331.1550 (2.16)	Illioliganones K (**13**) or E (**14**)	[31,33]
8	7.547	C_21_H_32_O_10_	[M + NH_4_]^+^ (462.2341)	133.0937 (100.00), 229.1092 (1.91), 287.1129 (1.83), 341.1190 (2.30), 409.1432 (0.48). 462.2341 (0.31)	Henrylactone D (**15**)	[19]
9	8.773	C_27_H_34_O_11_	[M + NH_4_]^+^ (552.2448)	137.0586 (88.95), 177.0886 (12.94), 237.1098 (20.37), 305.1507 (100.00), 355.1507 (100.00), 552.2448 (0.59)	Dihydrodehydrodiconiferyl alcohol 9-*O*-*β*-D-(3′-*O*-acetyl)-xylopyranoside (**16**)	[21]
10	8.855	C_27_H_34_O_11_	[M + NH_4_]^+^ (552.2448)	137.0522 (98.74), 177.0799 (16.41), 237.0996 (25.60), 305.1004 (33.79), 355.1346 (100.00), 415.0753 (1.05), 552.2448 (8.59)	Illiciumlignan F(**17**)	[34]
11	9.296	C_15_H_18_O_3_	[M + H]^+^ (247.1336)	146.0993 (100.00), 159.1164 (44.07), 173.1287 (58.84), 189.1254 (63.00), 213.1257 (21.07), 231.1357 (19.30), 247.1336 (22.20)	1-Allyl-2-(3-methylbut-2-enyloxy)-4,5-methylenedioxybenzene (**18**)	[32]
12	11.127	C_14_H_16_O_4_	[M + H]^+^ (249.1174)	145.0940 (87.48), 163.1009 (57.58), 175.1011 (39.15), 191.1021 (33.87), 203.0928 (19.31), 215.1304 (11.00), 233.1397 (25.03), 249.1174 (5.89)	Trans-3-methoxy-4,5-methylene-dioxycinnamaldehyde (**19**)	[29]
13	11.601	C_22_H_26_O_6_	[M + H]^+^ (387.1812)	225.0888 (4.39), 253.0858 (11.04), 281.0829 (13.40), 323.1258 (51.40), 355.1517 (100.00), 387.1812 (2.42)	Tashironin (**20**)	[19]
14	12.957	C_22_H_26_O_6_	[M + H]^+^ (387.1812)	167.0681 (7.82), 211.0714 (6.48), 263.1026 (9.50), 323.1217 (52.67), 355.1476 (100.00), 387.1812(4.11)	Illioliganfuranol A (**21**)	[33]
15	13.17	C_15_H_22_O_2_	[M + H]^+^ (235.1696)	137.0890 (34.98), 159.1094 (87.25), 165.1200 (27.72), 184.1164 (6.44), 199.1412 (100.00), 217.1527 (12.93), 235.1578 (8.33), 235.1696 (3.09)	(−)-1-Hydroxy-1,3,5-bisabolatrien-10-one (**22**)	[33]
16	13.301	C_22_H_24_O_6_	[M + H]^+^ (385.1654)	165.0563 (5.89), 225.0901 (7.64), 263.1055 (12.81), 295.1332 (32.59), 316.0933 (97.08), 353.1380 (100.00), 385.1654 (73.07)	4-Allyl-2,6-dimethoxyphenyl-3,4-dimethoxycinnamate (**23**)	[35]
17	14.788	C_14_H_22_O_2_	[M + H]^+^ (223.1698)	107.0796 (68.42), 121.0941 (100.00), 131.0833 (18.38), 147.1112 (83.63), 165.1207 (37.30), 178.0644 (8.49), 195.0740 (10.71), 210.0588 (5.76), 223.1698 (15.51)	1*β*-Isopropyl-4-β-methyl-9*β*-hydroxy spiro [4,5] dec-6-en-8-one (**24**)	[29]
18	14.919	C_15_H_24_O_2_	[M + H]^+^ (237.1852)	133.0998 (26.34), 147.1158 (100.00), 161.1307 (49.35), 179.1424 (27.87), 201.1602 (9.95), 219.1723 (28.61), 237.1852 (1.94)	10-Hydroxyacoronene (**25**)	[29]
19	15.05	C_22_H_30_O_6_	[M + H]^+^ (391.2118)	107.0435 (2.69), 145.0938 (5.85), 167.0635 (100.00), 205.1153 (59.58), 237.1415 (25.58), 391.2118 (1.55)	Tashironin A (**26**)	[30]
20	17.404	C_26_H_36_O_9_	[M + Na]^+^ (515.2284)	113.0543 (4.63), 184.0670 (2.57), 316.0877 (31.07), 385.1589 (100.00), 469.2170 (11.84), 469.2170 (11.84), 515.2284 (7.33)	(−)Secoisolariciresinol-*O*-*α*-L-rhamnopyranoside (**27**)	[36]
21	19.889	C_20_H_24_O_6_	[M + Na]^+^ (383.1475)	197.0903 (0.78), 225.0889 (2.61), 263.1051 (5.51), 314.0772 (98.58), 353.1369 (100.00), 368.1240 (1.84), 383.1475 (35.43)	(+)-Isolariciresinol (**28**)	[36]
22	21.85	C_10_H_12_O_3_	[M + Na]^+^ (203.1733)	105.0678 (100.00), 119.0832 (81.51), 133.0982 (43.59), 147.1136 (60.78), 161.1287 (20.74), 175.1407 (2.70), 203.1733 (12.13)	2-(4-Hydroxyphenyl) ethyl acetate (**29**)	[29]
23	23.550	C_15_H_22_O	[M + H]^+^ (219.1746)	109.1006 (100.00), 145.0999 (18.70), 173.1304 (5.94), 207.0295 (3.52),219.1746 (3.31)	*α*-Santal-11-en-10-one (**30**)	[37]
24	24.727	C_7_H_6_O_2_	[M-H_2_ + H]^+^ (121.1019)	102.0493 (2.04), 103.0555 (48.44), 105.0687 (100.00), 107.0820 (5.07), 115.0484 (3.19), 119.0838 (16.97), 121.1019 (14.47)	Hydroxybenzaldehyde (**31**)	[32]

## Supplementary Materials

The following are available online at https://www.mdpi.com/2076-3921/8/10/446/s1, Table S1. Primers used for qRT-PCR, Figure S1. The total ion chromatogram of EEIH in positive ion mode.
