# Supplementary material for: Hepatoprotective Effect of the Ethanol Extract of Illicium henryi against Acute Liver Injury in Mice Induced by Lipopolysaccharide
Source: Antioxidants (Basel). 2019 Oct 1;8(10):446. doi: 10.3390/antiox8100446 (PMC6826918; doi:10.3390/antiox8100446)
Supplement: Supplementary file 1 [file antioxidants-08-00446-s001.pdf]

Supplementary Materials

Table S1. Primers used for qRT-PCR.

Gene	Primer Sequence	Product Size (bp)
TNF-α	5′-CCACCACGCTCTTCTGTCTAC-3′<br>5′-GAGGGTCTGGGCCATAGAA-3′	104
IL-6	5′-ACAACCACGGCCTTCCCTACTT-3′<br>5′-CACGATTTCCCAGAGAACATGTG-3′	129
IL-1β	5′- GTAATGAAAGACGGCACACC -3′<br>5′- CTTTGGGTATTGCTTGGGAT -3′	98
iNOS	5′-GGCAGCCTGTGAGACCTTTG-3′<br>5′-GCATTGGAAGTGAAGCGTTTC-3′	72
COX-2	5′- GCAGATGACTGCCCAACTC-3′<br>5′- CAGGGATGAACTCTCTCCGT-3′	103
TLR4	5′-GTTGCAGAAAATGCCAGGATG-3′<br>5′-CAGGGATTCAAGCTTCCTGGT-3′	101
NF-κB p65	5′-ACGACATTGAGGTTCGGTTC-3′<br>5′-ATCTTGTGATAGGGCGGTGT-3′	124
Nrf2	5′-AGCCAGCTGACCTCCTTAGA -3′<br>5′-AGTGACTGACTGATGGCAGC-3′	131
GAPDH	5′-ATCCTGTAGGCCAGGTGATG-3′<br>5′-TATGCCCGAGGACAATAAGG-3′	113

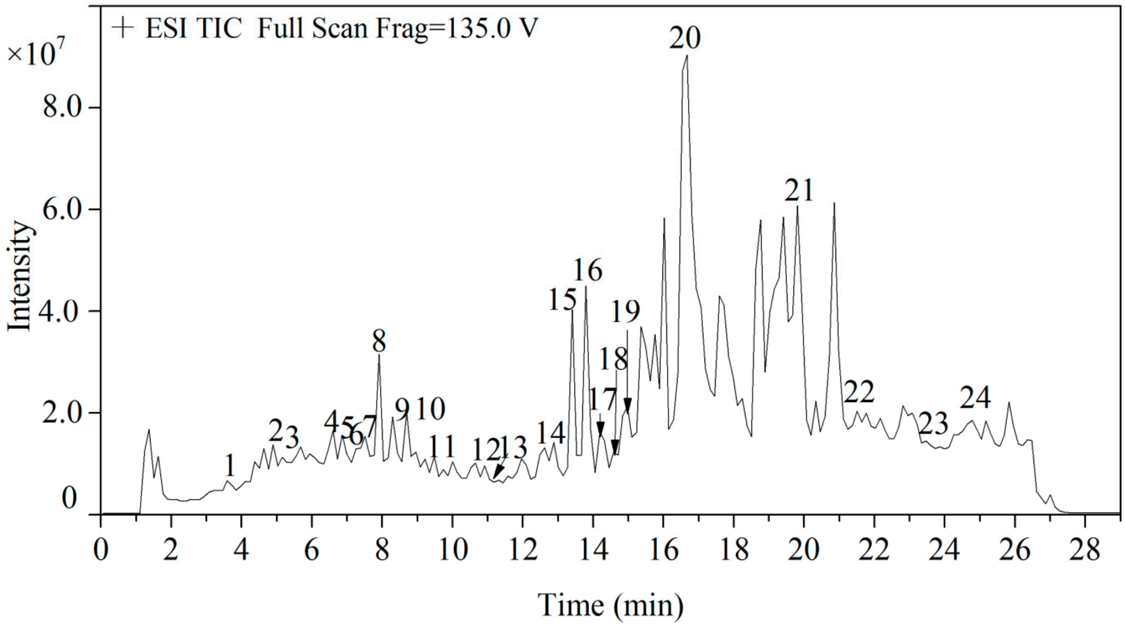

Figure S1. The total ion chromatogram of EEIH in positive ion mode.
